# Immune response of adults with secondary immunodeficiency to pediatric *Haemophilus influenzae* type b (Hib) vaccine

**DOI:** 10.1186/1710-1492-7-S2-A23

**Published:** 2011-11-14

**Authors:** Marina Ulanova, Nicole Hawdon, Eli Nix, Garry Ferroni, William McCready

**Affiliations:** 1 Northern Ontario School of Medicine, Thunder Bay, Ontario, Canada

## Background

Patients with chronic renal failure (CRF) develop secondary immunodeficiency as a result of the uremic state and its metabolic consequences, along with a negative impact of the dialysis procedure on the immune system. Such patients are therefore at high risk for septicemia and other severe infections. In non-vaccinated adults, protection against *Haemophilus influenzae* type b (Hib) is mediated by natural anti-capsular polysaccharide antibody. We hypothesized that non-vaccinated adults with CRF lack protective antibody against Hib, but may respond to the pediatric Hib vaccine.

## Methods

Serum anti-Hib IgG and IgM were studied in 60 patients with CRF and 40 healthy controls. Thirty-two patients and 19 controls were immunized with one dose of pediatric Hib vaccine; serum antibody levels were assessed pre- and 1, 6, 9 months post-vaccine. Functional antibody activity was studied using a serum bactericidal assay.

## Results

Almost 90% of controls, but only 43% of non-vaccinated CRF patients had protective anti-Hib antibody. Four week post-vaccination, all but one patient (97%) have developed protective antibody with a 14-fold increase (P<0.05); in 29 out of 32 (91%) the antibody exhibited bactericidal activity. In the majority of patients, protective antibody persisted 9 months post-vaccine. The vaccine response did not depend on the age, but was lower in CRF patients who had type 2 diabetes, COPD, or heart disease, compared to the rest of the group.

## Conclusion

Most adult patients with CRF are at an increased risk of acquiring invasive Hib disease as they lack protective antibody. The pediatric Hib vaccine is highly immunogenic in this group, with higher response compared to other vaccines administered to such patients (hepatitis B and pneumococcal vaccines). This study provides rationale for the immunization of individuals with secondary immnunodeficiency against Hib to achieve protective immunity.

